# Efficacy and Adverse Events of Carboplatin Desensitisation Therapy for Gynaecological Cancer: A Retrospective Study

**DOI:** 10.3390/medicines9040026

**Published:** 2022-03-30

**Authors:** Akihito Yamamoto, Seiryu Kamoi, Shigeru Matsuda, Rieko Kawase, Kazuho Nakanishi, Shunji Suzuki

**Affiliations:** Department of Obstetrics and Gynaecology, Nippon Medical School, 1-1-5 Sendagi, Bunkyo-ku, Tokyo 113-8602, Japan; skamoi@nms.ac.jp (S.K.); m-shigeru@nms.ac.jp (S.M.); s2021@nms.ac.jp (R.K.); knakanishi@nms.ac.jp (K.N.); shun@nms.ac.jp (S.S.)

**Keywords:** carboplatin, desensitisation, hypersensitivity, malignancy, gynaecology, therapeutic effects

## Abstract

Background: Carboplatin, the key drug used in treating gynaecological cancer, has an approximately 12–16% risk of hypersensitivity reactions. We aimed to investigate the efficacy and adverse effects of carboplatin desensitisation therapy for gynaecological cancer. Methods: The desensitisation protocol was standardised as a four-step, 4-h, carboplatin administration in the hospital. A retrospective medical record review was conducted on 15 patients who underwent carboplatin desensitisation for gynaecological malignancies at our hospital. Patients’ data were analysed to evaluate the treatment success rate, therapeutic effect of desensitisation, adverse events, and treatment. Results: Of 91 carboplatin desensitisation cycles scheduled; the completion rate was 93.4% (85/91). Adverse events occurred in 23 of these 91 (25.3%). In four (4.4%) of the 23 cycles, hypersensitivity reactions could be treated only by discontinuing the infusion and slowing the administration, while in the remaining 19 (20.9%), medication was administered intravenously after discontinuing the infusion to manage hypersensitivity reactions. No treatment-related deaths occurred. Overall, 23 series of anti-cancer agent regimens, including carboplatin desensitisation, were administered to the 15 patients. The therapeutic response rate was 82.6% and the disease control rate was 95.7%. Conclusions: Carboplatin desensitisation was beneficial in patients with a history of carboplatin-induced hypersensitivity reactions.

## 1. Introduction

Platinum agents are some of the most effective drugs for the treatment of gynaecological malignancies including ovarian, cervical, and endometrial cancers. Carboplatin, one of the platinum agents, is used most frequently. Compared to cisplatin, carboplatin demonstrates the same therapeutic efficacy with fewer adverse events (e.g., nausea, vomiting, auditory toxicity, nephrotoxicity) [1,2]. Platinum-taxane combination therapy is used as a neoadjuvant and adjuvant therapy in patients with gynaecological cancer [1,2,3]. In addition, many patients with recurrent gynaecological cancer receive carboplatin re-administration because studies have shown that there is an improvement in overall survival if there is an interval of 6 months or more between the end of platinum therapy and recurrence [4,5,6,7]. However, platinum agents can induce hypersensitivity reactions (HSRs). There is also a positive correlation between the risk of HSRs and the total number of administered carboplatin cycles [8,9,10]. HSRs caused by carboplatin have a probability risk of 12% to 16% [8,9].

The clinical symptoms of platinum HSRs range from mild to severe and death can also occur [11,12,13]. Mild reactions include skin manifestations, such as rash and itching; and gastrointestinal symptoms, such as stomach-ache and diarrhoea. Severe reactions include cardiovascular symptoms, such as hypotension, respiratory symptoms, such as hypoxia and bronchospasm, and anaphylaxis [14]. Approximately 50% of carboplatin hypersensitivity reactions (CHRs) are mild [9]. However, when patients undergo another carboplatin cycle, even patients with a history of mild HSRs can develop severe HSR, and symptoms often appear early [9,15].

HSRs to key drugs are problematic in some cases because of the lack of alternative therapies. Management options include enhanced premedication with antihistamines and corticosteroids, the discontinuation of carboplatin and switching to another drug, and carboplatin desensitisation (CD).

The goal of desensitisation is for patients to react less strongly to chemotherapeutic agents. In some reports, CD was successful in patients who experienced platinum-related HSRs [16,17,18,19,20,21]. Although the reports showed differences in the specific methods of desensitisation, almost all patients were able to complete the chemotherapy re-administration as planned [16,17,18,19,20,21]. Following a previous report, patients with CHRs in our institution underwent carboplatin re-administration.

The purpose of this study was to summarise the clinical features, toxicity, and effects of CD therapy on therapeutic efficacy, and outline possible appropriate prophylaxis and management strategies for treating the adverse events.

## 2. Materials and Methods

### 2.1. Patients

We retrospectively evaluated the experience of CD in patients with gynaecological malignancies who experienced HSRs with previous chemotherapy. Our study was conducted at a teaching hospital attached to a university medical school, a general hospital with approximately 900 beds. This study was approved by the Institutional Review Board of Nippon Medical School (No. 30-01-1067). We reviewed all patients who underwent CD between January 2015 and December 2019. In this retrospective review study, only patients who underwent CD following careful consultation were included. That is, among all gynaecological cancer patients, only those who had developed HSRs because of carboplatin administration and who consented to the risk of undergoing desensitisation were included. Patients who developed HSRs to carboplatin and who consented to CD but for whom detailed information on HSRs was not available, and patients who had not completed an anti-cancer agent treatment cycle, including CD at our hospital, were excluded. Due to the nature of the disease, all subjects were female. Initial HSR symptoms varied and included, cutaneous symptoms (vascular oedema, flushing, rash with or without itching); respiratory symptoms (bronchospasm, decreased oxygen saturation, cough, pant, chest tightness); cardiovascular symptoms (tachycardia, cold sweat, hypotension); gastrointestinal symptoms (nausea, vomiting, diarrhoea, stomach-ache); and atypical symptoms (limb dysesthesia and discomfort). Patients with adverse events associated with carboplatin administration were examined by oncologists. If the oncologist determined that their symptoms were carboplatin-induced HSRs, they were offered treatment alternatives, including CD therapy, another anti-cancer agent therapy, or best supportive care only. All patients understood the risks, including death, as well as the benefits of re-medication from the description of CD therapy, and they signed written informed consent for desensitization therapy. Consent to use the participants’ clinical information was obtained in writing and consent limited to this study was obtained by an opt-out method. After applying the inclusion and exclusion criteria, 15 patients with gynaecological malignancies (median age, 62 years; range, 43–73 years) subsequently opted for CD at our hospital. None of the patients underwent treatment after developing HSRs at another hospital or underwent an intradermal reaction test for carboplatin.

### 2.2. Desensitisation Protocol

The desensitisation protocol followed at our institution was standardised as a four-step, 4-h, carboplatin administration based on the report by Takase et al. [22] That is, the total amount of carboplatin was divided into four steps and administered over 4 h. This protocol was created based on the four-step 6-h protocol of Confino-Cohen et al. and the 12-step 3.8-h protocol of Lee et al. and is a practical method that requires a small number of steps and a short administration time [17,18]. However, when adverse events did occur, carboplatin administration is suspended and then slowed and resumed; therefore, not all desensitisation schedules are completed in 4 h. We made minor modifications to the protocol. The most important improvement was that the administration of the combined anti-cancer agents, other than carboplatin, was completed the day before CD, which was started early the following morning. The purpose of this arrangement was to avoid the risk of a night-time infusion of undiluted solution that was most likely to cause patients to re-develop HSRs. In addition, the protocol strictly stipulated the interruption of administration in the event of an adverse reaction and the slowing-down method, after its resumption. All patients were hospitalised for treatment and stayed overnight after desensitisation; patients were discharged the day after desensitisation if no adverse events occurred. Patients were treated in a room near the staff station, and emergency medications and equipment were provided in the room for immediate response. The doctor-in-charge waited in the ward until the administration of carboplatin was completed.

Under this desensitisation protocol, four different dilutions of carboplatin solutions were administered to patients over 1 h each. The total dose of carboplatin (AUC5-6 according to the regimen) was calculated using the Calvert formula. First, the total dose of carboplatin was dissolved in 250 mL of 5% glucose. Next, 25 mL of the solution were removed from the first solution and diluted with 225 mL of 5% glucose to obtain the second solution. This operation was repeated in sequence to prepare 1/10, 1/100, and 1/1000 diluted solutions. In other words, 250 mL of 0.1% solution, 225 mL of 1% solution, 225 mL of 10% solution, and 225 mL of 100% solution were prepared. Each solution was infused over 1 h, and all infusions were completed over 4 h. Prior to desensitisation, all patients were intravenously treated with glucocorticoids (dexamethasone 19.8 mg), H2 antagonists (famotidine 20 mg), H1 antagonists (chlorpheniramine 5 mg), and 5-HT3 antagonists (palonosetron 0.75 mg) (Table 1).

### 2.3. Management of Adverse Events

If HSRs re-emerged during desensitisation, carboplatin administration was immediately discontinued, and the patient was examined by the attending doctor. After confirming the improvement of the symptoms, the patient was allowed to resume the treatment 30 min later. When the administration was resumed, it was re-initiated at half the infusion rate at the time of discontinuation, and if no abnormalities were observed for 30 min, the infusion rate was restored to the original rate. If a mild reaction occurred, careful observation of the symptoms was performed without medication. When a moderate reaction occurred, an H1 antagonist (chlorpheniramine 5 mg), an H2 antagonist (famotidine 20 mg), and glucocorticoid (hydrocortisone 100 mg or methylprednisolone 125 mg) were administered intravenously. In addition, a phosphodiesterase inhibitor (aminophylline 250 mg) was used to treat respiratory symptoms. For severe reactions or anaphylaxis, an additional 0.3 mg of adrenaline was administered in addition to the glucocorticoid. The gynaecological oncologist in charge decided whether to continue or discontinue the desensitisation depending on the symptoms of CHRs. If treatment continuation was an option, the patient was verbally consulted, and consent obtained. Even if the doctor-in-charge determined that continuous administration was possible, the administration was discontinued according to the patient wishes. Desensitisation was discontinued in all cases of severe hypersensitivity and anaphylaxis.

### 2.4. Evaluation and Statistical Analysis

The clinical-stage was evaluated based on the staging criteria of the International Federation of Gynaecology and Obstetrics. Therapeutic effects were evaluated according to the Revised Response Evaluation Criteria in Solid Tumours guidelines (version 1.1) [23]. Adverse events were analysed using the National Cancer Institute’s Common Terminology Criteria for Adverse Events (CTCAE) (version 5.0) [24]. For the grade of adverse events, refer to Table 2.

The primary endpoint was the success rate of CD administration, which was calculated as the percentage of patients who completed all planned CDs among all patients and the percentage of completed CD cycles among the total CD cycles. The secondary endpoint was the response rate of the anti-cancer agent therapy, including CD. The treatment response rate was calculated as the percentage of consecutive treatments in which tumour shrinkage was observed in all consecutive treatments, including CD. An analysis using descriptive statistics was performed. The survey results were described and evaluated in terms of range, median, percentage, and so forth.

## 3. Results

### 3.1. Patients

Patient characteristics are shown in Table 3. Postoperative histopathological examination revealed that 14 patients were diagnosed with ovarian or primary peritoneal cancer (serous carcinoma, endometrioid carcinoma, mixed carcinoma, and unclassified carcinoma), and one patient was diagnosed with endometrial cancer (endometrioid carcinoma). Eleven patients (73.3%) had a history of allergies to various medications or foods. One patient experienced an initial CHR in adjuvant therapy and 14 patients experienced it during relapse treatment. The median number of carboplatin cycles preceding the initial HSR was 10 (range, 7–35), and the median cumulative dose was 5100 mg/m^2^ (range, 2240–12,000 mg/m^2^). The initial HSR grades ranged from 1 to 3 (grade 1, *n* = 4; grade 2, *n* = 0; and grade 3, *n* = 11).

The chemotherapy regimens used for desensitisation were paclitaxel + carboplatin (*n* = 20), paclitaxel + carboplatin + bevacizumab (*n* = 30), docetaxel + carboplatin (*n* = 6), docetaxel + carboplatin + bevacizumab (*n* = 8), gemcitabine + carboplatin (*n* = 19), and gemcitabine + carboplatin + bevacizumab (*n* = 8).

### 3.2. The Success Rate of Desensitisation

In total, the second and subsequent episodes of HSRs were observed during 23 cycles in eight patients. The details of each HSR episode and treatment results are shown in Table 4. In the first cycle of CD, 12 of the 15 patients (80%) were asymptomatic and successfully treated, but three experienced adverse events. Two of these three patients exhibited pruritic skin rashes and paraesthesia during the infusion (one while receiving the 1/10 diluted solution and the other while receiving the undiluted solution). These symptoms were alleviated immediately after the infusion was suspended, and the infusion was resumed without the need for pharmaceutical intervention for adverse events. These patients eventually completed the first desensitisation cycle. Another patient with adverse events developed palmar flushing and swelling of the lips during the infusion of the undiluted solution; therefore, hydrocortisone was administered intravenously after the carboplatin infusion was suspended. The patient did not require adrenaline because there were no respiratory or circulatory abnormalities. The patient improved only after suspending the infusion and administering hydrocortisone. Following this result, the doctor discussed the risk of continuing CD with the patient, and the patient eventually chose to discontinue the remaining treatments.

A total of 91 CD cycles were performed, 68 of which (74.7%) were completed without adverse events. In 23 (25.3%) of the CD treatments, patients developed some CHRs, but symptoms were relieved by a temporary suspension of administration or additional medication; among those, 17 CD cycles were completed as planned. As a result, 85 CD cycles (93.4%) were completed as originally planned.

Seven patients (46.7%) did not develop CHRs. Eight patients (53.3%) developed CHRs during the CD. Among them, three patients (20.0%) completed the scheduled treatment with only temporary interruptions or some changes in medication, while five patients (33.3%) discontinued the CD after consulting with their doctor. Treatment was not discontinued due to adverse events other than CHR or tumour exacerbation during the course of the treatment.

### 3.3. Antitumour Effect of Treatments Series, including CD

After completing the series of regimens including CD, the therapeutic effect was determined by comparing tumour image measurements before and after treatment. A total of 23 series of anti-cancer agent regimens, including CD, were administered to the 15 patients. Results were evaluated according to the Response Evaluation Criteria in Solid Tumours (version 1.1) as complete response (CR) 15 series, partial response (PR) 4 series, stable disease (SD) 3 series, and progressive disease (PD) 1 series. The response rate [(CR + PR)/(CR + PR + SD + PD)] was 82.6%, and the disease control rate [(CR + PR + SD)/(CR + PR + SD + PD)] was 95.7%. The average platinum-free interval before the start of the anti-cancer treatment series, including the CD regimen, was 17.8 months (range, 6–33 months) in 22 series, excluding one series of adjuvant therapy. Comparing the median platinum-free interval by treatment result, 14 series of CR was 19.5 (6–33) months, 4 series of PR was 14.5 (10–29) months, 3 series of SD was 10.0 (7–12) months, and 1 series of PD was 24 months.

### 3.4. Adverse Events and Treatments

Adverse events were observed in 23 of the 91 total CD cycles (25.3%). In 4 (4.4%) of these cycles, CHRs could be treated only by discontinuing the infusion and slowing down the administration. In the remaining 19 cycles (20.9%), medication was administered intravenously after the discontinuation of the infusion to manage CHRs.

Cutaneous adverse events were most common. Skin rash and itching were observed in seven patients; they were alleviated by discontinuation of CD or administration of additional drugs (grade 1 or grade 3). The next most commonly observed adverse events were those affecting the gastrointestinal system, such as stomach-aches and diarrhoea. Two patients developed symptoms and were treated with CD discontinuation and administration of H1 or H2 blockers (grade 1 or grade 3). One patient experienced grade 3 anaphylaxis (hypotension) but recovered quickly after discontinuing the carboplatin infusion, without requiring adrenaline and with no sequelae. Another patient experienced grade 3 anaphylaxis (bronchospasm and hypoxia) but quickly recovered with the addition of medications containing adrenaline. No treatment-related deaths occurred.

## 4. Discussion

We reported that carboplatin could be re-administered to patients who experienced CHRs, using the CD protocol. The therapeutic effect was satisfactory, and the recurrence of adverse events was in an acceptable range. This result is of great importance for patients; it stresses that prolonging their lives through additional carboplatin is feasible.

Platinum agents, such as carboplatin, are essential for the treatment of gynaecological malignancies, especially ovarian cancer. However, multiple doses of carboplatin can cause HSRs, and abandoning subsequent platinum administration and switching to platinum-free anti-cancer therapy is of great detriment to a patient’s prognosis. CHRs usually appear in 8–19% of patients receiving repeated doses of six cycles or more (eight times on average) [8,9,25]. CHRs are thought to be IgE mediated [14,19]; and strengthening premedication is one of the countermeasures, but premedication cannot completely prevent severe allergic reactions.

Previously, unsuccessful attempts to re-administer the drug that caused HSR using premedication alone have been reported [26]. On the other hand, Zorzou et al. attempted slow delivery in addition to premedication and succeeded in re-administering carboplatin but reported adverse events in 77% of patients [27]. HSRs have been reported with all platinum agents, but some reports state that cisplatin and nedaplatin are the best alternative agents for patients with a history of CHR [28,29,30,31,32]. In contrast, reports show that patients treated with cisplatin died after developing CHRs [11,12]. Moreover, there are reports of repeated HSRs with cisplatin and nedaplatin after developing CHRs [33,34]. Therefore, it is not always safe to change the type of platinum agent used. In addition, in these reports, the response rate of alternative platinum agents was inferior to that of carboplatin re-administration. Small initial doses with gradual escalation are thought to slowly consume IgE antibodies, and thus, avoid acute reactions. By extending the infusion time, the anti-cancer agent antigen is also delivered at a reduced concentration per unit time, thus reducing exposure to the antigen. This method has been reported to have relatively good results in recent years and is considered to be an effective re-administration method for patients who have experienced CHR [13,16,17,18,19,20,21,22,35,36,37,38,39]. However, the effectiveness of the protocol still relies on the reports of each institution because it is difficult to plan a prospective, multicentre study to determine a standard protocol.

In this study, we adopted a four-step, 4-h CD protocol in the hospital. The advantages of this protocol include its simplicity, short infusion times, and acceptable HSR rates [22]. The CD treatment was completed in 5 h, including premedication, and complete desensitisation was achieved during the day. We chose in-hospital CD treatment rather than outpatient treatment because we anticipated that some patients may need to suspend and resume deceleration, which may extend the time required for infusion and necessitate night-time administration. However, opinions regarding the place of administration are not unified. In a large series of case studies reported by Castells et al., a 6-h, 12-step desensitisation was first performed within the intensive care unit [19]. Subsequent desensitisation was performed in an inpatient or outpatient fluid centre, depending on the tolerability. However, patients with mild to moderate HSR do not need to be treated in the same strict environment as patients with severe HSR. In a four-step desensitisation protocol, Li et al. showed that outpatient desensitisation was possible in patients with a history of mild to moderate HSR [40]. In their study, HSR recurred in 32.6% of patients, but the symptoms were mild, and the treatment completion rate was 99%.

The majority of our patient cohort completed the CD without experiencing serious adverse events. In our study, HSRs occurred in 25.3% of the CD cycles, which is close to the incidence of 25.0% reported in the largest desensitisation study by Altwerger et al. [13]. Overall, 93.4% of the planned desensitisation cycles could be completed, resulting in a tumour control rate of 95.7%, allowing patients to obtain at least one therapeutic effect from CD. This result indicates that CD is a satisfactory treatment option, but it is not possible to evaluate the quality of the results in comparison with the reports of various oncologists. This is because the indication criteria and eligibility of HSRs have a strong influence on the success rate of desensitisation. We evaluated HSRs using the National Cancer Institute’s CTCAE v.5.0 [24]. Although these criteria are widely used worldwide, they are not suitable criteria for assessing CHRs. All HSRs that occurred in the patients in this study were classified as grade 2 when using the grading of infusion-related reactions. Therefore, HSRs were evaluated using the allergic reaction, but even for extremely mild hypersensitivity reactions, intravenous intervention is often performed to avoid the development of more serious symptoms; conversely, it is rare to choose the only oral intervention. As a result, grading generally corresponds to either follow-up (grade 1) or intravenous intervention (grade 3). To improve the existing grading system, which obscures the assessment of HSR severity, Brown et al. proposed a new assessment method, emphasising the importance of hypotension, hypoxia, and central nervous system symptoms in HSR grading [41]. Li et al. also proposed a severity classification based on the presence or absence of symptoms in at least one of the three systems of the cardiovascular, respiratory, and central nervous systems, as well as the duration of those symptoms. They performed desensitisation only in patients with mild to moderate HSRs, resulting in a high completion rate of 99% [40]. The patients in this study, including those who developed either hypotension or hypoxia due to CD, showed the same allergic symptoms in the initial CHR. Conversely, the patients without cardiovascular or respiratory symptoms at the initial CHR did not develop any of these symptoms. These results suggest that the type of HSR that occurs in CD may be very similar to that of the initial HSR. Attempting to limit the entry criteria for CD to mild to moderate initial CHR symptoms may help increase the success rate of CD and provide safer treatment.

Reviewing our results, discontinuation of CD was limited to 6 CD cycles, with 93.4% of cycles ending successfully. However, five patients did not complete the targeted number of CD cycles. Of the five dropouts, three were discontinued at the patient’s request. Strict adherence to our protocol rules meant that two patients failed CD, for a success rate of 86.7%. These two patients had initial CHRs with respiratory and circulatory symptoms and were at risk of developing severe adverse events during CD. As mentioned previously, we speculate that more stringent eligibility criteria for CD and exclusion of these two patients would have resulted in a higher success rate of desensitization, consistent with that reported by Li et al. [40].

The discontinuation criteria in this study were not clear, and the decision to discontinue the CD depended on the judgment of the attending doctor, which could obscure the results of the analysis. In our study, two patients with CHR that affected their hemodynamic status and respiratory function discontinued CD and did not resume the subsequent CD cycle because of the strong expectation of more serious life-threatening adverse events. On the other hand, the three patients with milder symptoms also discontinued the subsequent CD cycle after consulting with their doctors, considering that the purpose of treatment for recurrent cancer was not curative. Lee et al. and Castells et al. allowed resumption of subsequent CD, even in patients who required adrenaline, corticosteroids, or oxygen, but Takase et al. did not [17,19,22]. The treatment completion rate in this study was numerically similar to those of previous reports, but no meaningful comparison could be made without a common criterion for the discontinuation of CD.

Apart from the ambiguity of patient eligibility criteria and treatment discontinuation criteria mentioned above, the limitations of our study are its small sample size and retrospective nature. Because this was a retrospective study, patient characteristics were uncontrolled and varied in disease, stage, and the number of treatments. Therefore, in the future, it will be necessary to carefully consider the adaptation and discontinuation criteria, develop and comply with a protocol with unified standards, and conduct a large-scale investigation. In addition, the fact that male patients are not included in this study limited to gynaecologic cancers, will limit the versatility of this protocol. Although there are successful reports of CD on other types of cancer patients, that included males, using a protocol similar to ours, the findings derived from our study are limited to females [37,42]. As another limitation, the CD regimen used in this study varied in the type of anticancer drug administered in combination with carboplatin. However, we believe that anticancer drugs combined with the key drug carboplatin are very unlikely to be related to the outcome of recurrence and control of HSRs. The reason for this is that, due to our regimen arrangement, those drugs were used on separate days from carboplatin.

In conclusion, our findings suggest that CD is a good option for patients with a history of platinum-sensitive gynaecological cancer and CHR. CD carries some risk, but strict adaptation and discontinuation criteria may contribute to risk reduction. In addition, our improved protocol, which secures a day dedicated to carboplatin administration only, and which strictly regulates a slow administration, as a re-administration method after CHR, made it possible to administer CD more safely than the conventional protocols.

## Figures and Tables

**Table 1:** Carboplatin desensitisation protocol.

Premedication
1. Dexamethasone 19.8 mg in 50 mL NS infused over 30 min
2. Famotidine 20 mg, chlorpheniramine 5 mg, and palonosetron 0.75 mg in 50 mL NS infused over 30 min
**Carboplatin Desensitisation**
1. 1/1000 dilution in 250 mL 5% glucose infused over 60 min
2. 1/100 dilution in 225 mL 5% glucose infused over 60 min
3. 1/10 dilution in 225 mL 5% glucose infused over 60 min
4. Carboplatin AUC 5 or 6 in 225 mL 5% glucose infused over 60 min

NS: normal saline, min: minutes, AUC: area under the curve.

**Table 2:** Grading system for allergic reaction and anaphylaxis with Common Terminology Criteria for Adverse Events version 5.0.

	Allergic Reaction	Anaphylaxis
Grade	Clinical symptoms	
0	No reaction	No reaction
1	Systemic intervention not indicated	-
2	Oral intervention indicated	-
3	Bronchospasm: hospitalisation indicated for clinical sequelae; intravenous intervention indicated	Symptomatic bronchospasm, with or without urticaria, parenteral intervention indicated: allergy-related oedema/angioedema, hypotension
4	Life-threatening consequences: urgent intervention indicated	Life-threatening consequences: urgent intervention indicated
5	Death	Death

**Table 3:** Patients Characteristics.

Patient No.	Age (Year)	Allergy History	Diagnosis	FIGO Stage	Histologic Diagnosis	No. of Carboplatin Cycles until Initial CHR	Initial CHR Symptoms	Grade at Initial CHR
1	62	-	Ovarian cancer	IIIc	Endometrioid carcinoma G3	9	Whole body rash	3
2	43	Soybean	Ovarian cancer	IIIc	Serous carcinoma	8	Cold sweat, nausea, itching	3
3	71	Iodine contrast agent	Endometrial cancer	Ia	Endometrioid carcinoma G2	10	Whole body rash, itching	3
4	62	Metal	Ovarian cancer	IIIc	Endometrioid carcinoma G3	12	Conjunctival hyperaemia, nausea	3
5	58	Iodine contrast agent, Rebamipide, Loxoprofen Na	Ovarian cancer	IVb	Mixed carcinoma (endometrioid and serous)	10	Rash on limbs, itching, abdominal discomfort	3
6	64	Loxoprofen Na, Fexofenadine hydrochloride	Primary peritoneal cancer	IIIa	Adenocarcinoma	35	Palmar rash, facial flushing, itching	3
7	57	House dust	Ovarian cancer	IIIc	Serous carcinoma	9	Itching	3
8	45	-	Ovarian cancer	IVb	Serous carcinoma	15	Facial flushing, itching, stomach-ache, vomiting, diarrhoea, cold sweat	3
9	66	Pregabalin, Rebamipide, Loxoprofen Na	Ovarian cancer	IIIc	Serous carcinoma	7	Arm rash	1
10	53	-	Ovarian cancer	IIIc	Serous carcinoma	9	Whole body rash	1
11	73	Iodine contrast agent, Blue fish	Ovarian cancer	IIIc	Serous carcinoma	12	Body rash	3
12	69	Pollen	Ovarian cancer	IIIc	Serous carcinoma	28	Bronchospasm, hypoxia, dyspnoea, whole body rash	3
13	59	Almond, Cherry, Pineapple	Ovarian cancer	IIIc	Serous carcinoma	23	Hypotension, cold sweat	1
14	57	Shrimp, Squid	Peritoneal cancer	IIIc	Serous carcinoma	16	Palmar rash, facial flushing	1
15	63	-	Peritoneal cancer	IVb	Serous carcinoma	8	Whole body rash	1

No., number; FIGO, International Federation of Gynaecology and Obstetrics; CHR, carboplatin hypersensitivity reaction; G, grade.

**Table 4:** Result of CD.

Treatment *	Patient No.	Regimen	PFI	Response ^†^	CD Cycles	CHR	Grade	Treatment for G3 CHR	Result
1	1	TC + Bev	9	CR	3	Rash, itching	G0 × 1, G1 × 2	N/A	Completion × 3
2	1	TC + Bev	7	SD	6	Rash, itching	G0 × 3, G3 × 3	Hydrocortisone	Completion × 6
3	2	TC	10	PR	4	N/A	0	N/A	Completion × 4
4	3	TC	21	CR	2	N/A	0	N/A	Completion × 2
5	4	TC + Bev	26	CR	4	N/A	0	N/A	Completion × 4
6	4	DC + Bev	17	PR	8	Facial flushing, rash, itching, stomach-ache, nausea, diarrhoea, cold sweat, dyspnoea, discomfort	G0 × 3, G3 × 5	Chlorpheniramine, famotidine, methylprednisolone	Completion × 7, Discontinuation × 1
7	5	DC	24	CR	6	N/A	0	N/A	Completion × 6
8	6	GC	13	CR	4	N/A	0	N/A	Completion × 4
9	6	GC	9	CR	6	N/A	0	N/A	Completion × 6
10	7	TC	18	CR	3	N/A	0	N/A	Completion × 3
11	7	TC	12	PR	2	Rash, itching	G0 × 1, G3 × 1	Chlorpheniramine, hydrocortisone	Completion × 1, Discontinuation × 1
12	8	TC + Bev	23	CR	3	Stomach-ache, itching	G0 × 1, G1 × 2	Chlorpheniramine, hydrocortisone	Completion × 3
13	8	TC + Bev	15	CR	6	Stomach-ache, itching	G1 × 6	Chlorpheniramine	Completion × 6
14	9	GC + Bev	28	CR	2	N/A	0	N/A	Completion × 2
15	9	GC	17	SD	5	N/A	0	N/A	Completion × 5
16	9	GC + Bev	7	SD	3	N/A	0	N/A	Completion × 3
17	10	TC + Bev	33	CR	2	Rash, conjunctival hyperaemia	G0 × 1, G3 × 1	Chlorpheniramine	Completion × 1, Discontinuation × 1
18	11	TC + Bev	28	CR	4	N/A	0	N/A	Completion × 4
19	12	TC + Bev	15	CR	2	Bronchospasm, hypoxia, cough, facial flushing	G0 × 1, G3 × 1	Chlorpheniramine, famotidine, hydrocortisone, adrenaline	Completion × 1, Discontinuation × 1
20	13	GC + Bev	29	PR	3	N/A	0	N/A	Completion × 3
21	13	GC	24	PD	4	Hypotension	G0 × 3, G3 × 1	Hydrocortisone	Completion × 3, Discontinuation × 1
22	14	TC	6	CR	8	N/A	0	N/A	Completion × 8
23	15	TC	N/A	CR	1	Rash	G3	Hydrocortisone	Discontinuation × 1

CD: carboplatin desensitisation, No.: number, PFI: platinum free interval, CHR: carboplatin hypersensitivity reaction, TC: paclitaxel + carboplatin, Bev: bevacizumab, DC: docetaxel + carboplatin, GC: gemcitabine + carboplatin, CR: complete response), SD: stable disease, PR: partial response, PD: progressive disease, G: grade. * A series of consecutive anti-cancer drug treatments for each patient was counted as one treatment. Treatments for the same patient but not consecutive were counted as different treatments. ^†^ The effects of continuous treatment, including the CD cycles, were evaluated in total.

## Data Availability

All data generated or analysed during this study are included in this article. Further enquiries can be directed to the corresponding author.
